# Ultrasensitive Determination of Natural Flavonoid Rutin Using an Electrochemical Sensor Based on Metal-Organic Framework CAU−1/Acidified Carbon Nanotubes Composites

**DOI:** 10.3390/molecules27227761

**Published:** 2022-11-11

**Authors:** Yuhong Li, Jianxiong Tang, Yueli Lin, Jiejun Li, Yaqi Yang, Pengcheng Zhao, Junjie Fei, Yixi Xie

**Affiliations:** 1 Key Laboratory of Environmentally Friendly Chemistry and Applications of Ministry of Education, College of Chemistry, Xiangtan University, Xiangtan 411105, China; 2 Key Laboratory for Green Organic Synthesis and Application of Hunan Province, Xiangtan University, Xiangtan 411105, China; 3 Hunan Institute of Advanced Sensing and Information Technology, Xiangtan University, Xiangtan 411105, China

**Keywords:** electroanalysis, pyrolysis, carbon materials, drug analysis, modified electrode

## Abstract

Rutin, a natural flavonol glycoside, is widely present in plants and foods, such as black tea and wheat tea. The antioxidant and anti-inflammatory effects of flavonoids are well known. In this study, a new electrochemical rutin sensor was developed using multiwalled carbon nanotubes/aluminum-based metal–organic frameworks (MWCNT/CAU-1) (CAU−1, a type of Al-MOF) as the electrode modification material. The suspension of multiwalled carbon tubes was dropped on the surface of the GCE electrode to make MWCNT/GCEs, and CAU−1 was then attached to the electrode surface by electrodeposition. MWCNTs and CAU−1 were characterized using scanning electron microscopy (SEM) and X-ray photoelectron spectroscopy (XPS). Due to the synergistic effect of CAU−1 and MWCNT-COOH, the prepared sensor showed an ultrasensitive electrochemical response to rutin. Under optimized conditions, the sensor showed a linear relationship between 1.0 × 10^−9^~3.0 × 10^−6^ M with a detection limit of 6.7 × 10^−10^ M (S/N = 3). The sensor also showed satisfactory stability and accuracy in the detection of real samples.

## 1. Introduction

Rutin, a natural flavonoid glycoside, is widely found in citrus fruits, vegetables, herbs, and plant-derived beverages [1]. It is well known as an antioxidant with versatile health benefits, including anti-inflammation, antiaging and antimicrobial effects, disease-related enzyme inhibition, vascular endothelial protection, and so on [2,3,4]. Accurate determination of rutin is important in evaluating its pharmaceutical value and potential risks of overconsumption [5]. Recently, electrochemical sensors for natural flavonoids, including rutin, have attracted much interest because of their advantages of being simple, fast, efficient, and low-cost [6]. The traditional detection techniques are quantification using gas chromatography, high-performance liquid chromatography and fluorescence spectrometry, and capillary electrophoresis [7,8,9]. In recent years, electrochemical sensor detection technology has been widely studied because of its ease of operation, high sensitivity, and ability to detect low concentrations [10,11]. Since the electrochemical sensor is usually based on an electrode whose detection performance is closely related to the modified material, researchers have devoted themselves to employing different nanomaterials to construct rutin electrochemical sensors [12]. Nanomaterials with good conductivity or electrocatalytic activity, such as precious metals, carbon materials, metal oxides, conductive polymers, and bimetallic nanoparticles and their composites, have often been investigated [13,14,15,16]. Furthermore, materials with molecular recognition functions, such as enzymes, cyclodextrin, and molecularly imprinted material, have usually been used as a unit to enhance the selectivity of the sensors [17,18]. However, materials that are both low cost and have high selectivity and sensitivity are still scarce [19]. Therefore, it is still necessary to develop high-performance rutin electrochemical sensors with new functional materials.

Metal–organic frameworks (MOFs) have attracted great interest in recent decades [20]. They are three-dimensional porous materials that use metal ions or cationic metal clusters as coordination nodes to bridge various organic ligands, and the metal cation nodes and organic ligands are the most basic building blocks of MOF crystals [21]. Through layer-by-layer self-assembly, metal–organic framework materials with various geometric topologies can be synthesized [22]. The tunable structures, large specific surface areas, and highly ordered pore structures make them favored materials in the field of catalysis, energy storage and transfer, gas adsorption and separation, and chemical sensing [23,24,25]. For electrochemical sensing, MOFs also have great potential because of the possible electrocatalytic capacity endowed by the unsaturated metal coordination sites, high concentrate and mass transfer efficiencies caused by the abundant pores and large specific surface area, and good selectivity toward specific analytes due to the size exclusion effects of the specific channels and cavities [26].

Some MOFs have been employed to construct rutin electrochemical sensors; conductive materials have usually been introduced to make up for the weak conductivity of MOFs [27,28,29]. CAU−1 is an aluminum-based MOF with high porosity and thermal stability that contains octameric building blocks connected by amino terephthalate linkers [30]. CAU-1 has been used in the fields of seawater desalination, drug delivery, gas separation, catalysis, etc., but it has rarely been investigated in applications in electrochemical sensing [31,32,33,34]. In fact, within the molecular structure of CAU−1, there are a large number of -OH and -NH_2_ groups, which can form hydrogen bonds with the target analyte [32]. Moreover, aluminum, as a metal node, can catalyze the analyte, and the interior of CAU−1 is a porous structure with a large specific surface area that can adsorb several target analytes [35]. These properties endow CAU-1 with great potential to electrochemically sense molecules with phenolic hydroxyl groups. Sim et al. modified a GCE with CAU-1 to detect hydroquinone in tap water, obtaining a detection limit of 0.067 μm under optimized conditions [36]. Hence, more CAU−1 composite-based electrochemical sensors with better performance are worth studying further.

MWCNTs are conductive materials with excellent electrochemical catalytic activity and biocompatibility and are often used in electrochemical sensors to increase the overall conductivity and catalytic properties of the material [37,38]. Feng Gao et al. prepared multiwalled carbon nanotubes (ZIF-L) decorated with two-dimensional leafy zeolite imidazolate frameworks (MWCNT/ZIF-L) using a facile solvent method and used them as electrode materials for thiabendazole (TBZ)-sensitive electrochemical sensing [39]. Qin et al. developed an electrochemical sensor based on zeolitic imidazolate framework-8 (ZIF-8) polyhedron/multiwalled carbon nanotube (MWCNT)-modified electrodes for detecting rutin in medical tablets [40]. Zhiwei Lu et al. developed a high-performance electrochemical sensor prepared by continuous alternating electrodeposition of MWCNT-COOH and Zr-metal organic skeletons (UiO-66-NH_2_); this sensor was eventually used as a high-performance electrochemical sensor for the simultaneous determination of Cd^2+^ and Pb^2+^.

**Scheme 1:**
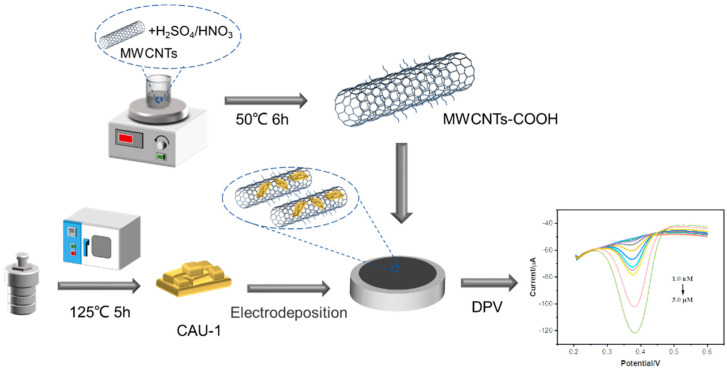
Illustration of the formation of MOF CAU−1 and the analytical principle of the electrochemical sensor based on CAU−1/MWCNT-COOH/GCE.

Here, considering that rutin contains polyhydroxy groups that can form hydrogen bonds with CAU−1, a CAU−1-based electrochemical sensor for rutin was proposed. Carboxylic acid functionalized multiwalled carbon nanotubes (MWCNT-COOH) were added to form a composite with better conductivity and stability than individual CAU−1. The composite was characterized and utilized to modify GCE to construct a rutin sensor. The preparation process of the sensor is shown in Scheme 1. Due to the synergistic effect of CAU−1 and MWCNT-COOH, the prepared sensor showed an ultrasensitive electrochemical response for rutin and could be applied in the actual sample analysis.

## 2. Results and Discussion

### 2.1. Characterization

**Figure 1:**
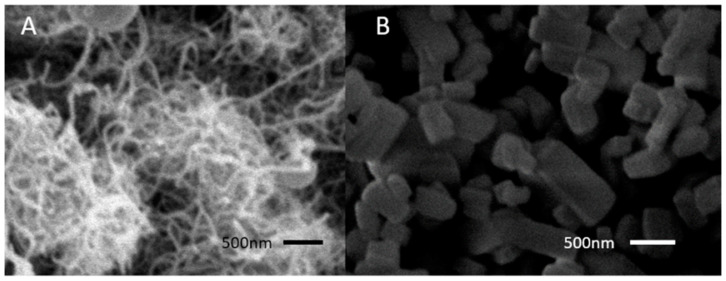
SEM images of (**A**) MWCNTs and (**B**) CAU−1.

To characterize the surface morphology of the electrode modification materials, an SEM scan was performed. As shown in Figure 1A, the MWCNTs showed a crisscrossed and evenly distributed network of fiber nanostructures, and the tube diameter was between 20 and 40 nm. Figure 1B is the SEM image of CAU−1; it can be seen that it was a smooth surface cuboid particle, and the particle texture was uniform.

To determine the crystal structure of CAU−1 and MWCNTs, powder XRD was used to determine whether CAU−1 and MWCNTs were successfully synthesized. As shown in Figure S1A, the broad diffraction peak at 2θ = 25.74° corresponds to the crystal plane (002) of the MWCNTs. Figure S1B shows the polycrystalline structure of CAU−1, grown along the (011) direction at 2θ = 6.9°; another sharp but slightly less intense peak was observed at 2θ = 13.8°, corresponding to the (022) diffraction plane. In addition, a sharp peak was displayed at 2θ = 9.9°, and the direction was (002), indicating that the additionally filled CAU−1 crystals were bound at the -OH-terminated interface. These characterization data are consistent with the previous literature [30], which proves the successful synthesis of CAU−1.

Figure S2A is the XPS diagram of CAU−1, in which it can be observed that CAU−1 contains four elements: C, O, N, and Al. As shown in Figure S2B, the C1s peak can be decomposed into four peaks. The binding energies were 284.8 eV (C-C), 286 eV (C-O), 286.7 eV (C-N/C=N), and 288.9 eV (C=O), respectively. The N1s peak, as shown in Figure S2C, can be decomposed into three peaks of 398.55 eV (C=N), 399.54 eV (C-NH_2_), and 401.23 eV (C-NH^3+^), which explain the existence of the amino group inside the MOF. As seen in Figure S2D, the 2p peak of Al can be decomposed into two peaks of 73.76 eV and 74.41 eV, which correspond to the presence of Al-O and Al-OH groups. These data reveal that CAU−1 was successfully synthesized [35].

### 2.2. Electrochemical Responses of Different Modified Electrodes to Rutin

**Figure 2:**
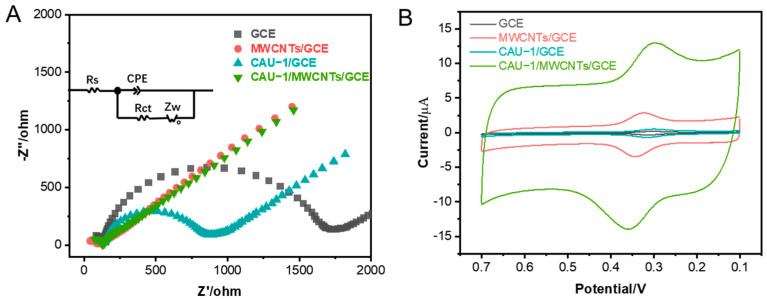
(**A**) EIS spectra of different electrode materials, supporting electrolyte solution: 1 mM Fe(CN)_6_^3−^/^4−^ (containing 0.1 M KCl) (inset: equivalent circuit diagram); (**B**) electrochemical responses of different electrode materials to 2 μM rutin at pH = 5.5.

According to the EIS results, the electron transfer impedance of the surface of the tested electrode was proportional to the curvature radius of the Nyquist curve [41]. The EIS impedance plots of the different modified electrodes are shown in Figure 2A and Figure S3. The electron transfer resistance (Rct) of the various modified electrodes was obtained from the figure and fitted and analyzed using the classical electrochemical model (Rs((Rct-Zw)-CPE)). As can be seen in Figure 2A, the conductivities of CAU−1/GCE (Rct = 712.2 Ω) and bare GCE (Rct = 1571 Ω) were poor. However, the corresponding Nyquist curves CAU−1/MWCNT/GCE and MWCNT/GCE were almost in a straight line, which indicates that the addition of MWCNTs significantly enhanced the electronic conductivity of the electrodes. These results indicate that MWCNTs not only act as good carriers but also effectively make up for the poor conductivity of CAU−1.

Figure 2B shows the electrochemical response of different electrodes to 2 μM rutin solution in a PBS solution (pH = 5.5). Among the three modified electrodes, CAU-1/GCE had the lowest redox peak current due to its low electron conduction efficiency, while the current value of CAU-1/MWCNT/GCE was greater than that of MWCNT/GCE, which was the highest among the three electrodes, indicating a synergistic effect between MWCNTs and CAU−1 [42]. Moreover, the CAU−1 was attached to the multiwalled carbon nanotubes by the method of electrodeposition, which effectively prevented carbon nanotubes from falling off the electrode. Since the -NH_2_ present in the CAU−1 ligand can interact with -OH in the rutin molecule to form a hydrogen bond, the surface of the CAU−1 modified electrode can specifically adsorb rutin molecules, which greatly enhances the detection signal. Furthermore, the catalytic functions of CAU−1 also can accelerate the redox process of rutin. In Figure 2B, it can be seen that the response of MWCNT/GCE was lower than CAU−1/MWCNT/GCE, indicating that the film formed by electrodeposition made the active site of CAU−1 well exposed. Hence, the CAU−1 with enrichment and catalysis functions might play a synergistic role with the highly conductive MWCNTs to allow CAU−1/MWCNT/GCE to obtain a sensitive detection signal for rutin.

### 2.3. Optimization of CAU-1/MWCNT/GCE Preparation

#### 2.3.1. Optimization of the Additional MWCNTs

To determine the optimal added concentration of MWCNTs, dispersions of MWCNTs of 0.4 mg mL^−1^, 0.6 mg mL^−1^, 0.8 mg mL^−1^, 1 mg mL^−1^, 1.5 mg mL^−1^, and 2 mg mL^−1^ were first prepared. The MWCNT-modified electrochemical sensor was added with 2 μM rutin in a 0.1 M PBS (pH 5.5) buffer solution to obtain rutin response peaks under different MWCNT concentrations. The results are shown in Figure S4A,C. As shown, when the concentration increased between 0.4 mg mL^−1^ and 1.0 mg mL^−1^, the signal of the modified electrode increased with the increase in MWCNT concentration and reached the peak at 1 mg mL^−1^. When the concentration continued to increase, the current response decreased with the increase in the concentration. This is because the performance of the electrode first increased with the concentration of the carbon tube due to its good conductivity and film-forming properties. However, when the concentration was too high, the carbon tube attached to the electrode surface was too thick, which not only hindered the electron transfer rate but also increased the drop of the MWCNTs from the electrode surface. Hence, 1 mg·mL^−1^ was selected as the optimal concentration of MWCNTs.

#### 2.3.2. Optimization of the Electrodeposition Turns of CAU−1

To determine the effects of CAU−1 electrodeposition turns, the performances of the sensors prepared under a series of deposition half cycles of 1, 3, 5, 10, and 15 were recorded separately. With other conditions and parameters unchanged, DPV was used to determine rutin in a 2 μM solution with the prepared electrodes. As seen in Figure S4B,D, before the number of half cycles reached 5, the peak current increased with the number of electrodeposition cycles, and after that, the peak current began to decrease gradually. Hence, the deposition half cycle of 5 was selected as the detection condition.

### 2.4. The Effect of pH on the Signal of Rutin

To explore the effect of different pH levels on the detection of rutin, the CV signals of the electrode in a PBS solution with 2 μM rutin at different pH levels (4.0, 4.5, 5.0, 5.5, 6.0, 6.5, and 7.0) were recorded. The potential range was set to 0.1–0.7 V, and the scan speed was 50 mV s^−1^. It can be seen in Figure S5A that the electrical signal gradually increased in the range of pH 4.0–5.5 and reached a peak at pH 5.5. After pH 5.5, the electrical signal gradually decreased with increasing pH. Therefore, pH 5.5 was chosen as the optimal detection condition. Furthermore, as the pH changed from 4.0 to 7.0, both oxidation and reduction potentials shifted negatively, indicating the involvement of protons in the electrochemical reaction of rutin [43]. It can be seen in Figure S5B that the change in the tested peak potential showed a linear relationship with the change in pH value. The linear relationship between anodic peak potential (E_pa_), cathodic peak potential (E_pc_), standard electrode potential (E^θ^), and PH was as follows:

E_pa_ = −0.0659pH + 0.7772 (R^2^ = 0.9951) E^θ^ = −0.0619pH + 0.7309 (R^2^ = 0.9986) E_pc_ = −0.0579pH + 0.6846 (R^2^ = 0.9988)

The slope of the equation for E^θ^ was close to −61 mV pH^−1^, which was close to the theoretical value of the Nernst equation (−59 mV pH^−1^); it can be concluded that the reaction of rutin on the electrode surface is a reversible process. The amount of electron gain and loss was the same as that of the proton transfer in the redox process of rutin on the surface of the electrode.

### 2.5. Influence of Scan Rate

Figure S6A shows the CV patterns of the electrode tested on the 2 μM rutin solution at different scan rates (5–280 mV s^−1^). It can be seen in Figure S6B that the currents of the oxidation peak and reduction peak had a good linear relationship with the scan rate, which was basically proportional. The linear relationship between the oxidation peak current (I_pa_) and reduction peak current (I_pc_) and sweep speed was as follows:

I_pc_ (μA)= 0.2401v (mV/s) − 2.8918 (R^2^ = 0.9992) I_pa_ (μA)= −0.2153v (mV/s) + 1.5771 (R^2^ = 0.9977)

Combined with the logarithmic plot of peak current versus scan rate (Figure S6C), both peak currents had slope values of 1.25 with values close to 1. It has been found that the electrochemical behavior of the electrode on rutin is controlled by adsorption [44,45]. It can also be observed in Figure S6A that the redox peak potential values under different scan rates were almost the same, with a small change range, and the positions of the oxidation peak and the reduction peak were almost the same, with only a small offset, so the electrode process is reversible. The graph is a reversible adsorption peak. With an increase in the scan rate, the reduction peak appeared after the diffusion wave and moved slightly to the negative potential to form a back wave because the adsorption capacity of the product (oxidized state) on the electrode surface was slightly stronger than that of the product (reduced state). In addition, for the reversible reaction, the number of electrons involved in the redox reaction of rutin was calculated as approximately 2 according to the equation proposed in the literature [46], and the possible reaction mechanism is shown in Figure S7.

### 2.6. Influence of Accumulation Potential and Accumulation Time

The effect of accumulation parameters was studied by changing the accumulation potential and accumulation time. The results are shown in Figure S8. Figure S8A is a line graph of the change in the peak current with the accumulation time. It can be seen in the figure that the peak current value was the highest at 350 s, the current increased with an increase in the enrichment time at 0–350 s, and the peak current remained constant with the increase in the enrichment time at 350–500 s. As Figure S8B shows, the peak current varied with the accumulation potential; it can be seen in the figure that before the accumulation potential was −0.1 V, the peak current value increased with the positive movement of the accumulation potential and reached the peak at −0.1 V. Then, as the potential continued moving forward, the value of the peak current gradually became stable or decreased. Therefore, the optimized accumulation time for the detection process is 350 s, and the optimized accumulation potential is −0.1 V.

### 2.7. Study on the Detection Performance of Modified Electrodes

**Figure 3:**
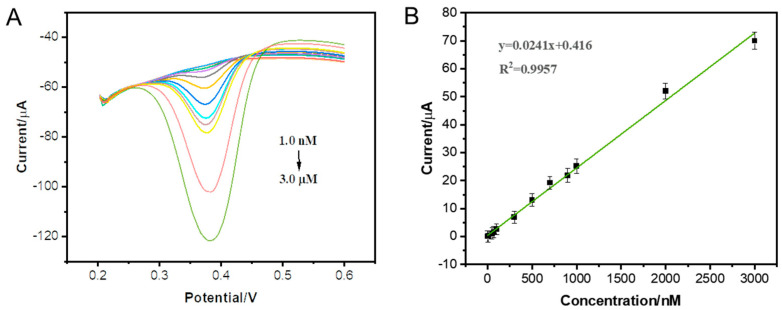
(**A**) DPV pattern of CAU−1/MWCNT/GCE at different rutin concentrations. (**B**) Working curve of CAU−1/MWCNT/GCE for rutin detection.

Differential pulse voltammetry (DPV) was used to study the rutin sensor performance under a scanning range of 0.2–0.6 V and a scanning speed of 50 mV/s. The chemical response results are shown in Figure 3A. As shown in Figure 3B, the electrical signal response of the modified electrode to rutin increased steadily with the increase in the concentration. The peak current and the concentration of rutin had a linear relationship between 1 × 10^−9^ and 3 × 10^−6^ M, and the linear equation was I_pa_ = 0.0241C(μM) + 0.416 (R^2^ = 0.9957); the detection limit was 6.7 × 10^−10^ M (S/N = 3), and it can be considered that the CAU-1/MWCNT/GCE-modified electrode can be used as a good sensor of rutin for quantification detection.

To evaluate the performance of the present sensor, some reported sensors for rutin are listed in Table 1 with details. Compared with the others, this sensor has higher performance and a lower detection limit; therefore, CAU−1/MWCNT/GCE has excellent detection performance for rutin.

### 2.8. Anti-Interference Detection

DPV was used to study the effect of interfering substances on the oxidation peak current of rutin (concentration: 1 μM), these substances were selected mainly on the basis of interaction type, functional group, and structural similarity. As shown in Figure S9, the error was within ±5%; about 50 times the concentration of K+, Fe^3+^, glucose (GLU), ascorbic acid (AA), and L-leucine (L-Leu) and 10 times the concentration of hesperidin (Hes) and lauric acid (LA) in PBS buffer solution and bisphenol A (BPA) had little effect on the detection of rutin, and the sensor had a good anti-interference ability.

### 2.9. Stability and Reproducibility

**Figure 4:**
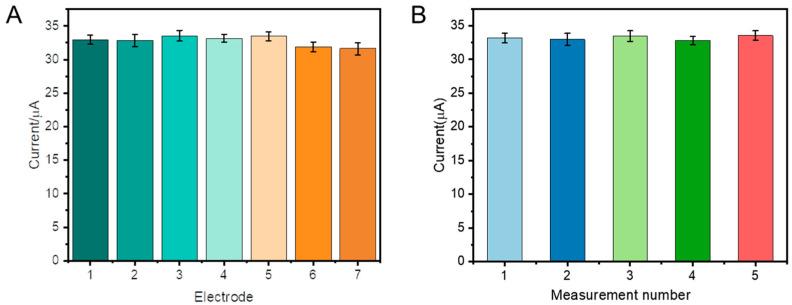
(**A**) DPV current values obtained by seven different electrodes in 1 μM rutin; (**B**) DPV current values obtained by five repeated tests in 1 μM rutin.

In the buffer solution of 0.1 M PBS (pH = 5.5) containing 1 μM rutin, the stability test of a single electrode was carried out by DPV technology, and the peak current signal decreased slightly with the increase in the number of scans. As shown in Figure 4A, after seven repeated experiments, the relative standard deviation of the seven oxidation peak currents was determined to be 6.95%. To evaluate the reproducibility of CAU-1/MWCNT/GCE, five independent electrodes were used to detect 1 μM rutin in a PBS solution, and the relative standard deviation (RSD) of the experiment was 2.78%, as shown in Figure 4B.

### 2.10. Detection in Real Samples

To verify the feasibility of the sensor platform for the detection of actual samples, the contents of rutin in commercial healthcare medical rutin tablets were detected. The rutin tablets were dissolved in ethanol and injected into a PBS solution (0.1 M, pH = 5.5), and the concentration of rutin in the solution was determined by the standard addition method. By adding different concentrations of the standard rutin solution, a series of determination results was obtained, as shown in Table 2. In the experiment, the recovery rate of the samples was 92–105.6%, and the standard deviation was 1.35–1.98%. These results show that our constructed CAU-1/MWCNT/GCE sensor platform can accurately detect the rutin concentration in real samples.

## 3. Materials and Methods

### 3.1. Reagents and Apparatus

Aluminum chloride hexahydrate (AlCl_3_·6H_2_O) was purchased from Aladdin Reagent Co., Ltd. (Shanghai, China). 2-Aminoterephthalic acid was obtained from Aladdin Reagent Co., Ltd. (Shanghai, China). Rutin was obtained from Yuanye Biotechnology Co., Ltd. (Shanghai, China). Hesperidin, glucose, ascorbic acid, L-leucine, and bisphenol A were purchased from Chengdu Purechem-Standard Co., Ltd. (Chengdu, China). Phosphate buffer solution (PBS) was obtained by mixing sodium dihydrogen phosphate (0.1 M NaH_2_PO_4_) and sodium monohydrate phosphate (0.1 M Na_2_HPO_4_). Commercial rutin tablets (produced by Shanxi Yunpeng Pharmaceutical Group Co., Ltd. (Linfen, China)) were purchased from the local pharmacy. All the solutions were prepared with deionized water.

The electrochemical measurements were recorded on a CHI-660E electrochemical workstation (Shanghai Chenhua, China). A platinum electrode was used as the counter electrode, and an Ag/AgCl electrode was used as the reference electrode. A bare glassy carbon electrode and modified electrodes were applied as working electrodes. All the electrodes were obtained from Chenhua Co., Ltd. (Shanghai, China). The characterizations of morphology and structure were performed by employing a Hitachi H7700 scanning electron microscope (Tokyo, Japan), a Thermo Fisher ESCALAB 250Xi X-ray photoelectron spectrometer (MA, USA), and a Philips X′pert Pro Super diffractometer.

### 3.2. Preparation of CAU−1

CAU−1 was prepared according to the previous literature with slight modifications [30]: aluminum chloride hexahydrate (2.967 g, 12.3 mmol) and 2-amino-terephthalic acid (0.746 g, 4.1 mmol) were dissolved in 30 mL of anhydrous methanol, and then the mixed solution was poured into a 50 mL Teflon-lined reactor heated at 125 °C for 5 h. After that, the precipitate was centrifuged, washed with methanol 3 times, and then soaked in deionized water for purification; this process was repeated 3 times at an interval of 6 h. The obtained materials were finally dried at 60 °C for 12 h under vacuum conditions to obtain dry CAU−1 particles in bright yellow. To prepare the electrodeposition solution, 20 mg of CAU−1 particles was dispersed in 10 mL of DMF solution with further addition of 15 mmol of NaCl and ultrasonically mixed at 25 °C for 2 h to obtain a CAU−1 solution (2 mg mL^−1^).

### 3.3. Preparation of MWCNT-COOH

The carboxylation of MWCNTs was carried out according to the literature with slight modifications [55]. First, 1.0 g of MWCNTs was added to a 100 mL mixed acid solution of concentrated nitric acid and concentrated sulfuric acid (volume ratio 1:3) and mixed at 50 °C for 6 h. The suspension was then diluted sixfold with deionized water and suction-filtered (0.42 aqueous filter membrane), and the filter residues were washed to neutrality with secondary deionized water. The residues were dried in an oven at 80 °C for 12 h to obtain black MWCNT-COOH powders. For GCE modification use, a 1 mg mL^−1^ MWCNT-COOH suspension was prepared by ultrasonically dissolving 3 mg of MWCNT-COOH powder into 3 mL of ultrapure water.

### 3.4. Preparation of CAU−1/MWCNT/GCE

A glassy carbon electrode (GCE) was used as the working electrode, which was polished with 0.3, 0.1, and 0.05 μm alumina powder in turn. After polishing, anhydrous ethanol and deionized water were used for ultrasonic cleaning to remove the residual alumina powder on the surface. The cleaned electrode was then dried under an infrared lamp before modifications. To prepare CAU−1/MWCNT/GCE, 6 μL of 1 mg mL^−1^ MWCNT-COOH was first dropped on the surface of GCE and then dried under the infrared lamp. Then, MWCNT-COOH/GCE was inserted into the 2 mg mL^−1^ CAU−1 solution, and the electrodeposition of CAU−1 was performed by 5 cycle scans with a cyclic voltammetry of 0.1 V s^−1^ in the potential range of −1.2~2.0 V. After drying, the proposed CAU−1/MWCNT/GCE was obtained. The specific steps are shown in Scheme 1.

### 3.5. Electrochemical Measurements

The potential range of cyclic voltammetry (CV) was set between 0.1–0.7 V, the scan rate was 50 mV/s, and the potential range of differential pulse voltammetry (DPV) was 0.2–0.6 V. The electrochemical impedance spectroscopy (EIS) scan was carried out in a 5.0 mM [Fe(CN)_6_]^4−^/^3−^ solution containing 0.1 M NaCl, in which the amplitude was set at 50 mV, a pulse width of 5 mV was selected, and the frequency range was between 100 kHz and 0.01 Hz.

## 4. Conclusions

In this paper, a novel metal–organic framework-based electrochemical sensing platform, CAU−1/MWCNT/GCE, was constructed by the electrochemical deposition method, and the detection of rutin in an aqueous solution was carried out. CAU−1 had a large specific surface area and abundant hydrogen bonding groups, such as -NH_2_ and -OH, and shows a high affinity for phenolic compounds. Its aluminum metal node also had a catalytic effect on the redox reaction. The addition of carboxylic MWCNTs improved the electron conduction efficiency of CAU−1 and enhanced the stability of the electrode. Under optimized conditions, CAU−1/MWCNT/GCE showed ultrasensitive performance for the detection of rutin, with a linear range of 1 nM–3 μM and a detection limit of 0.67 nM (S/N = 3). In addition, the sensor platform was successfully applied to the detection of real samples of rutin tablets in an aqueous solution. The sensor platform constructed by this method has high sensitivity and stability and good reproducibility.

## Figures and Tables

**Table 1:** Some previous work of electrochemical sensors for rutin.

Rutin Sensors	LOD	Linear Range	Detection Measures
AuNPs-HCNT/GCE [47]	81 nM	1.0 × 10^−7^–3.1 × 10^−5^ M	CV
PdPc-MWCNT/GCE [48]	75 nM	1.0 × 10^−7^–5.1 × 10^−5^ M	CA
N-MCS@GO/GCE [49]	50 nM	5.0 × 10^−7^–1.9 × 10^−4^ M	DPV
PtNP–rGO/GCE [50]	10 nM	5.0 × 10^−8^–1 × 10^−5^ M	DPV
Au–Ag nanorings/NG [51]	10 nM	5.0 × 10^−7^–2.4 × 10^−4^ M	DPV
[Mn^III^Mn^II^(Ldtb)(μ-OAc)_2_] BPh_4_ [52]	17.5 nM	9.99 × 10^−7^–6.54 × 10^−5^ M	SWV
rGO-InTAPc/GCE [53]	2.0 nM	5.0 × 10^−9^–1.0 × 10^−4^ M	DPV
PC/CoWO_4_/GCE [54]	0.45 ng/mL	5–5000 ng/mL	DPV
CAU−1/MWCNT/GCE (This work)	0.67 nM	1.0 × 10^−9^–3.0 × 10^−6^ M	DPV

AuNPs–HCNTs: gold nanoparticle-decorated helical carbon nanotube nanocomposites; PdPc–MWCNTs: palladium phthalocyanine-multiwalled carbon nanotube; CA: chronoamperometry; N-MCS: N-doped mesoporous carbon nanospheres; PtNP: platinum nanoparticle; NG: N-doped graphene; [Mn^III^Mn^II^(Ldtb)(μ-OAc)_2_] BPh_4_: mixed-valence diacetate-bridged manganese complex; SWV: square wave voltammograms; rGO-InTAPc: phthalocyanine indium-decorated reduced graphene oxide; PC: porous carbon.

**Table 2:** Detection in real samples.

	Original	Add	Detected	Recovery	RSD
1	49 nM	0	49 nM	---	1.56%
2	49 nM	25 nM	69 nM	92%	2.03%
3	49 nM	50 nM	101 nM	104%	1.35%
4	49 nM	75 nM	121 nM	96%	3.66%
5	49 nM	100 nM	148 nM	99%	2.63%
6	49 nM	125 nM	181 nM	105.6%	3.98%

## Data Availability

Data are contained within the article.

## Supplementary Materials

The following supporting information can be downloaded at: https://www.mdpi.com/article/10.3390/molecules27227761/s1, Figure S1: XRD patterns of (A) MWCNTs and (B) CAU−1; Figure S2: XPS survey of the CAU−1 (A) and high-resolution XPS spectra of (B) CAU−1-C1s, (C) CAU-1-N1s, (D) CAU−1-Al2p; Figure S3: EIS spectra of different electrode materials, supporting electrolyte solution: 1 mM Fe (CN)_6_^3−^/^4−^ (containing 0.1 M KCl); Figure S4: (A) Peak current values and (C) DPV current responses when the carbon tube concentrations were 0.2, 0.4, 0.6, 0.8, 1.0, 1.5, and 2.0 mg·mL^−1^. (B) The peak current values and (D) DPV current responses when the electrodeposition half-cycles were 1, 3, 5, 10, and 15; Figure S5: (A) Cyclic voltammetry curves of CAU−1/MWCNT/GCE at different pH levels (4.0, 4.5, 5.0, 5.5, 6.0, 6.5, and 7.0) to 2 μM rutin. (B) Linear relationship between pH and redox potential picture; Figure S6: (A) Different scan rates (at 5, 10, 20, 30, 40, 50, 60, 70, 80, 90, 100, 120, 140, 160, 180, 200, 220, 240, 260, and 280) of CAU−1/MWCNT/GCE in the presence of 2 μM rutin. (B) Linear relationship between scan rate and oxidation peak and reduction peak current; (C) Logarithm of peak current vs. logarithm of scan rate; Figure S7: The proposed mechanism of electrochemical oxidation of rutin. Figure S8: (A) Schematic diagram of peak current changing with accumulation time, (B) Schematic diagram of peak current changing with accumulation potential; Figure S9: Relative magnitude of electrochemical response signals of CAU−1/MWCNT/GCE to 1 μM rutin in the presence of different interfering substances in PBS solution.

## Sample Availability

Samples of the compounds are not available from the authors.
