# Supplementary material for: Ultrasensitive Determination of Natural Flavonoid Rutin Using an Electrochemical Sensor Based on Metal-Organic Framework CAU−1/Acidified Carbon Nanotubes Composites
Source: Molecules. 2022 Nov 11;27(22):7761. doi: 10.3390/molecules27227761 (PMC9695502; doi:10.3390/molecules27227761)
Supplement: Supplementary file 1 [file molecules-27-07761-s001.zip › molecules-2000430-supplementary.pdf]

Ultrasensitive determination of natural flavonoid rutin using an electrochemical sensor based on metal-organic framework CAU−1/acidified carbon nanotubes composites

Yuhong Li [1, §], Jianxiong Tang [1, §], Yueli Lin [1], Jiejun Li [1], Yaqi Yang [1], Pengcheng Zhao [2], Junjie Fei [1, 3, *] and Yixi Xie [1, 2, *]

[1] Key Laboratory of Environmentally Friendly Chemistry and Applications of Ministry of Education, College of Chemistry, Xiangtan University, Xiangtan 411105, People's Republic of China;

[2] Key Laboratory for Green Organic Synthesis and Application of Hunan Province, Xiangtan University, Xiangtan 411105, People's Republic of China;

[3] Hunan Institute of Advanced Sensing and Information Technology, Xiangtan University, Xiangtan 411105, People's Republic of China;

* Correspondence: Junjie Fei, fei_junjie@xtu.edu.cn; Yixi Xie, xieyixige@xtu.edu.cn.

§ These authors contributed equally to this work.

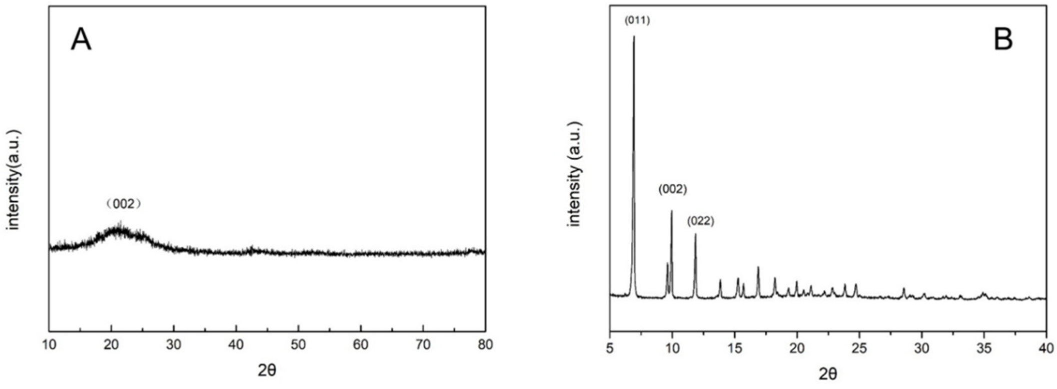

Figure.S1.XRD patterns of (A) MWCNTs and (B) CAU−1

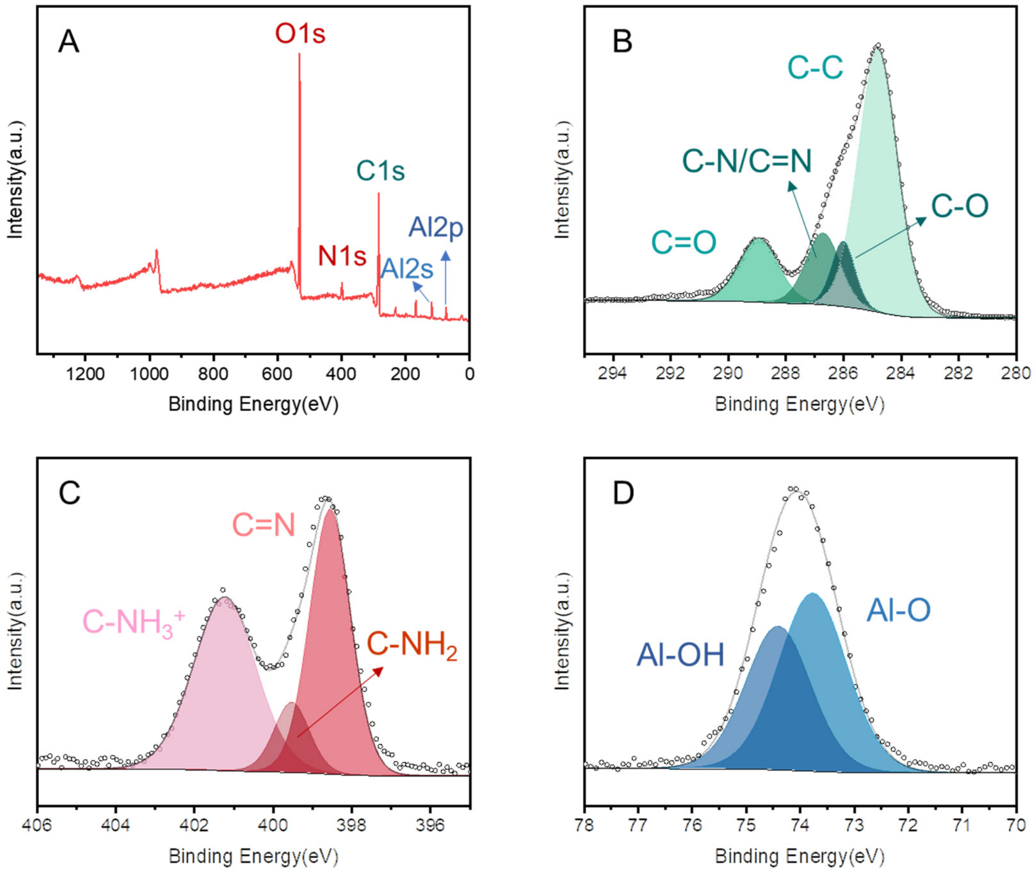

Figure.S2. XPS survey of the CAU-1(A) and High-resolution XPS spectra of (B) CAU-1-C1s, (C)CAU−1-N1s, (D)CAU−1-Al2p

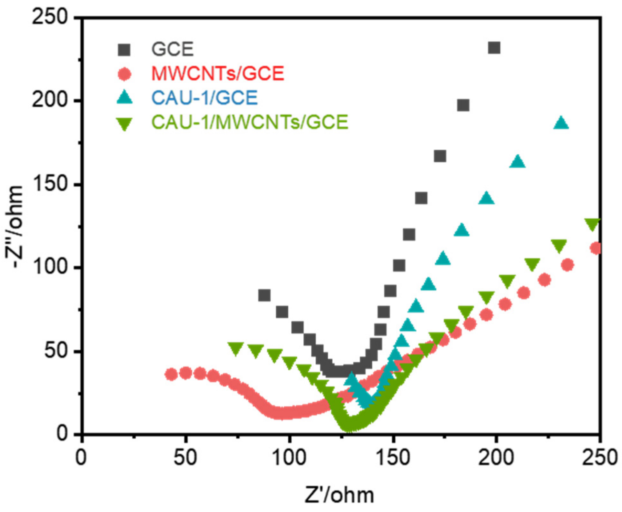

Figure.S3 EIS spectra of different electrode materials, supporting electrolyte solution: 1 mM Fe $(CN)_6^{3-/4-}$ (containing 0.1 M KCl)

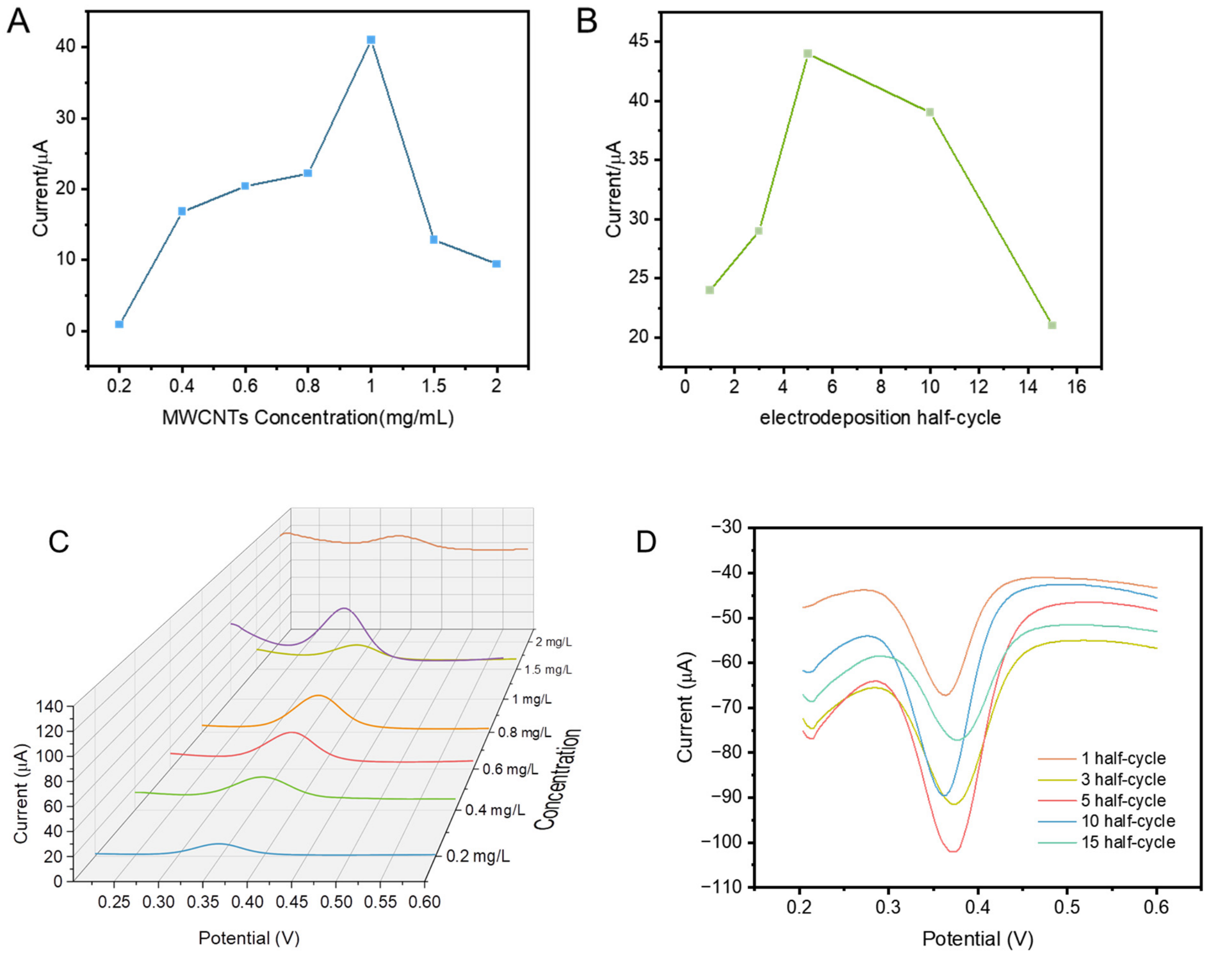

Figure.S4. (A) Peak current values and (C) DPV current responses when the carbon tube concentrations are 0.2, 0.4, 0.6, 0.8, 1.0, 1.5, and 2.0 mg•mL^{-1}. (B) The peak current values and (D) DPV current responses when the electrodeposition half-cycles are 1, 3, 5, 10, and 15.

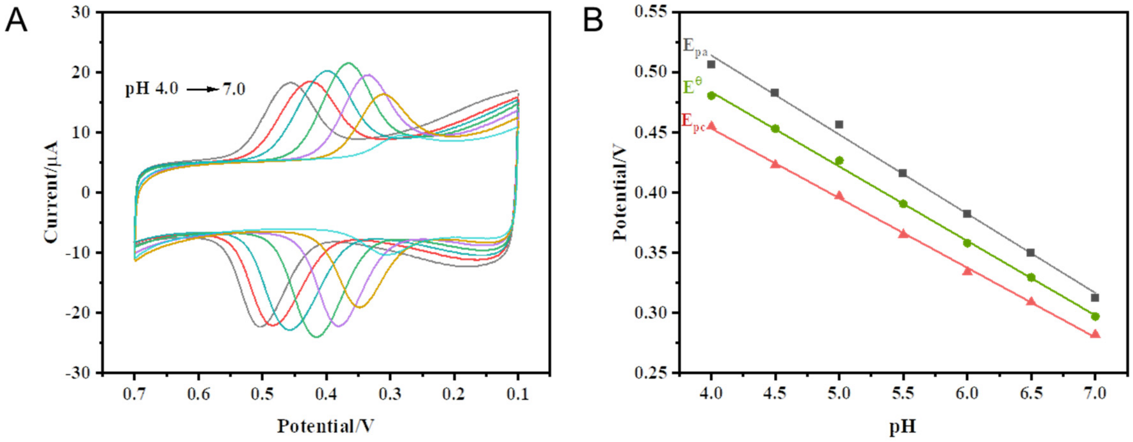

Figure.S5. (A) Cyclic voltammetry curves of CAU−1/MWCNTs/GCE at different pH (4.0, 4.5, 5.0, 5.5, 6.0, 6.5, 7.0) to 2 μM rutin. (B) Linear relationship between pH and redox potential picture

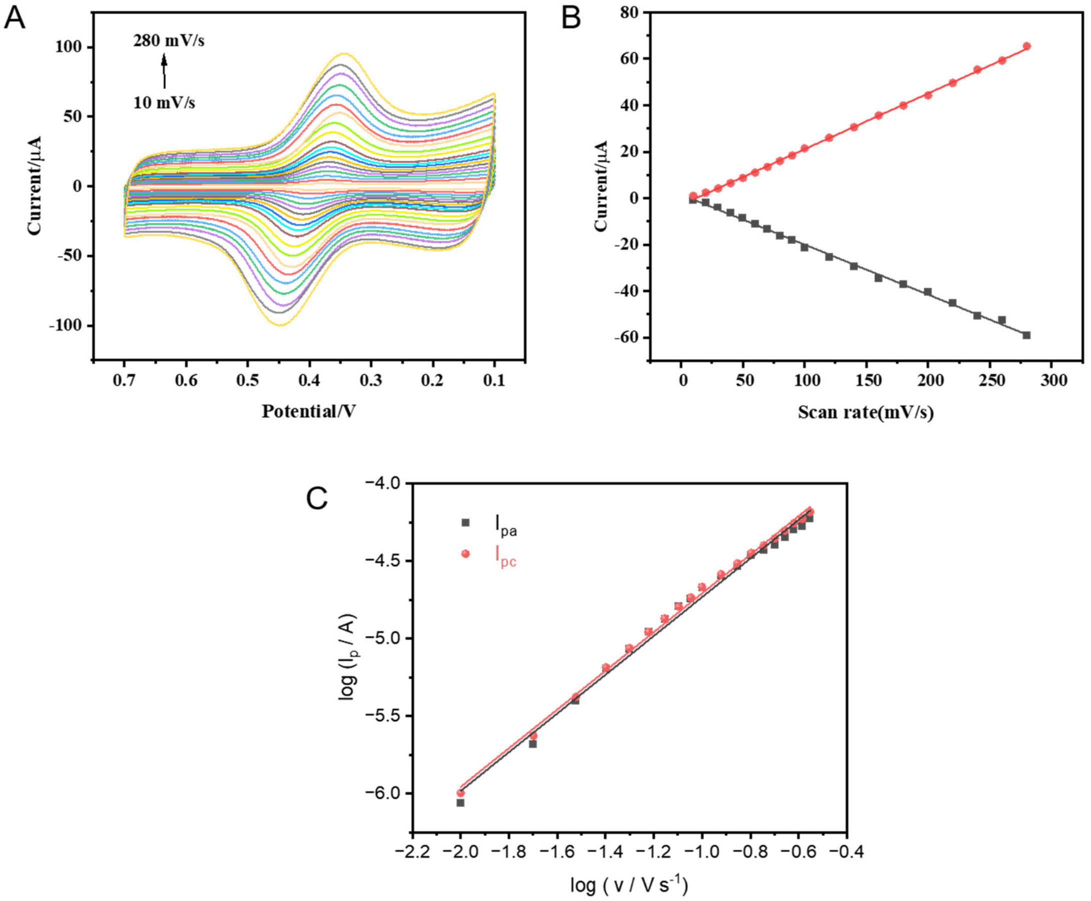

Figure.S6. (A) Different scan rates (at: 5, 10, 20, 30, 40, 50, 60, 70, 80, 90, 100, 120, 140, 160, 180, 200, 220, 240, 260, 280) of CAU-1/MWCNTs/GCE in the presence of 2 μM rutin. (B) Linear relationship between scan rate and oxidation peak and reduction peak current. (C) Logarithm of peak current vs. logarithm of scan rate

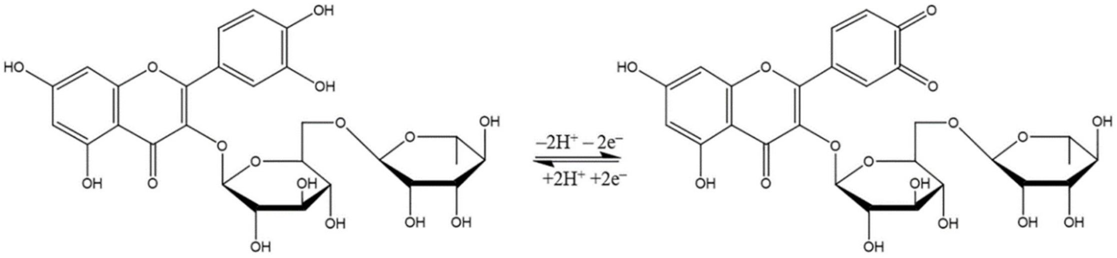

Figure.S7. The proposed mechanism of electrochemical oxidation of rutin.

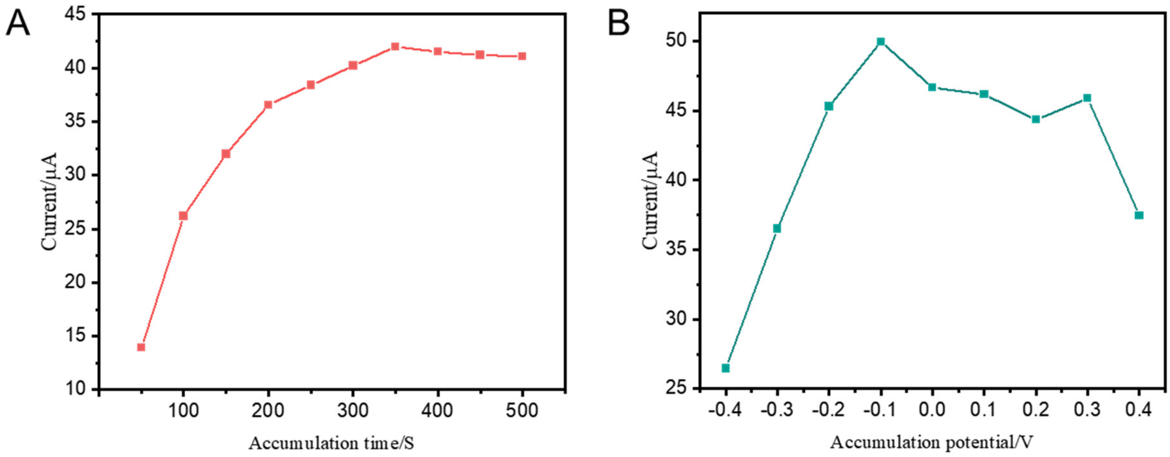

Figure.S8. (A) Schematic diagram of peak current changing with accumulation time (B) Schematic diagram of peak current changing with accumulation potential

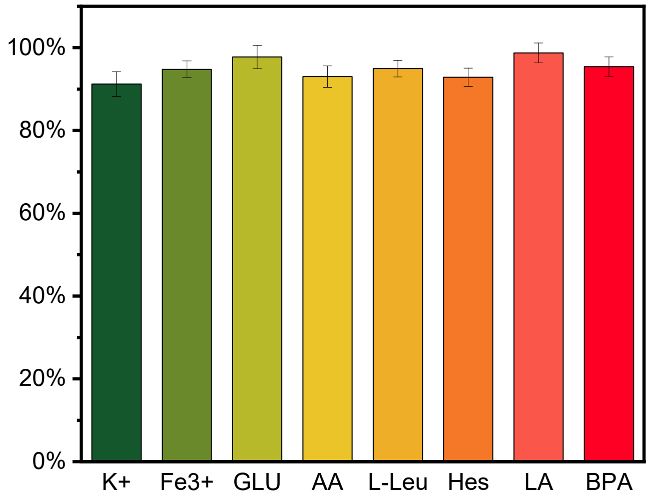

Figure.S9. Relative magnitude of electrochemical response signals of CAU−1/MWCNTs/GCE to 1 μM rutin in the presence of different interfering substances in PBS solution
